# The Proper Mission of the Obstetric Forceps

**Published:** 1885-10

**Authors:** S. F. Starley

**Affiliations:** Tyler, Texas, Ex-President Texas State Medical Association


### THE PROPER MISSION OF THE OBSTETRIC FORCEPS.

*By S. F. Starley, M. D., of Tyler, Texas, Ex-President Texas State Medical Association.*

For Daniel's Texas Medical Journal.

IT would seem that in this day of progress where so many sources of scientfic knowledge are open to the student and practitioner of the obstetric art, a proper knowledge of the various indications for the use of the forceps would be indellibly fixed in the mind of every man who takes upon himself the responsibile duties of the accoucheur. But unfortunately for humanity such is not the case. If we inquire of medical practitioners in general, especially in the country and smaller towns, we shall find that there are few, very few, who are in the habit of using the instrument at all. And why is this? It is because there is a widespread, but groundless, prejudice amongst the laity that instrumental interference with the process of labor is frought with danger to the mother and child; while too many physicians are ready to chime in with the ignorant old women and contend that nature should take its course in nearly every case. While the parturient woman is permitted to suffer on through untold agonies until her stock of vital force is exhausted, her blood poisoned with the effete material that is being carried into the vital fluid in great excess in consequence of the prolonged and fruitless effort that she is compelled to make for her delivery, her nervous energies become exhausted, and a feverish state of the system sets in, from which she is fortunate if she escapes without serious or permanent injury.

The indications for delivery with the forceps are more numerous than would be inferred from a perusal of the works of most authors on this subject. Prof. Maughs, certainly one of the ablest obstetric teachers in the United States, gives the following as the most common of the conditions requiring their employment:

"1st. Prolapse of the cord. 2nd. Hemorrhage. 3rd. Convulsions. 4th. Large head. 5th. Contracted pelvis. 6th. Unfavorable positions. 7th. Rigidity of os uteri. 8th. Rigidity of perineum. 9th. Feeble or ineffectual uterine contractions."

These certainly constitute the more frequent indications, and some one of them is generally found to be the cause of delay and danger to both mother and child, and in each of them, and the complications growing out of them, the forceps is the safest and most speedy means of relief at our command.

Prof. Maughs, speaking of rigidity of the perineum, and the delay and danger so frequently consequent upon that cause, says: "Of course, if delay occurs, here the forceps should be used—not after waiting until the head has rested immovable upon the perineum for six hours, as taught by Denman. Wait not six hours, nor one hour, but resort to the forceps as soon as it is manifest the head is not advancing, or, if advancing, doing so so slowly that it is evident the woman and child might suffer from unnecessary delay. Experience has taught what a *priori* might be inferred—that delays, at this point, are extremely dangerous to mother and child; dangerous to the mother because of the exceeding sensitiveness of the parts upon which the pressure is exercised; dangerous to the child because at this time it is mostly out of the womb, which is correspondingly contracted, interrupting the placental circulation. And to permit the occurrence of unnecessary delay here, would also be the less excusable, as the meerest tyro can apply the forceps at this point without difficulty or danger; and yet under the waiting-on-the-resources-of-nature doctrine, thousands of women have been permitted to endure the tortures of this condition for many weary hours, until their offspring had perished and their own lives endangered or sacrificed, when they could have been safely delivered in ten minutes. *O, tempora; O, mores!* Is the wail of the helpless woman of no monent? Of all our race are her sufferings alone to be disregarded, because, forsooth, there is some physiology in her sufferings?"

Then, speaking of feeble uterine contractions, the same learned teacher continues: "This is, perhaps, of all others, the most frequent cause of delay in labor, protracted second stage of labor through which the unfortunate, unaided woman drags through weary hours or days in fruitless effort to deliver herself, until the child is dead, and the mother in a hardly less unfortunate condition. In these cases the forceps reaches its highest triumph, and to withhold it, is a sin against God and humanity."

"When the gross clouds of ignorance, that have heretofore darkened our art, shall have given away to a knowledge of the mechanism of labor, the dangers of delay, and the ready and safe means of relief by making the forceps, a blessing, instead of a mockery to perturient women, such cases, with their dire consequences, will be unknown."

Speaking of its benefits and dangers, he says: "As it is the most conservative of obstetrical operations, always intended to benefit the mother and child, if this be living at the time, its dangers might almost be left out of the question, as its use necessarily involves absolutely no danger at all, except in the hands of the rash and ignorant, and such should never be at the bedside of the lying-in woman, and should they unfortunately get there, are as likely to do mischief without, as with the forceps." "Consequently as we are not talking to or writing for either the rash or ignorant, but to an educated profession where all are alike conscientious, and more or less skilled in the use of forceps, we may, as before stated, readily leave out its supposed or possible dangers—dangers far more *in posse* than *in esse*. And yet this is a very talisman for the trusting owls who have heretofore sat for hours by the bedside of a delicate, sensitive, helpless, suffering woman, in the the agonies of an unavailing labor, without giving her the unspeakable benefits of a speedy, safe and easy delivery by the forceps. Such a one may possibly console himself with the so oft repeated caveat for ignorance that "meddlesome midwifery is bad." So it is, but the indecision and hesitancy of fools is worse.

"But has it come to this, that the accoucheur is the only one of our divine profession who utterly ignores our lofty mission which is to save life and alleviate human suffering? And is her life and that of her unborn child of such non-importance that we are to turn a deaf ear to her prayer for help?" "The opponents of the early and frequent use of the forceps, refer with seeming triumph to the fact that in British midwifery practice one woman in every twenty deliveries with the forceps died; of course they did. They died, not because they were delivered with the forceps, but as we shall incontestably show, because they were not delivered earlier; they died because, through a slavish adherence to the authority of those whose ignorance and want of skill,—Denman & Co. should have prevented their ever being consulted, their labor was allowed to linger on until fatal inflammations were lighted up. They died because their blood was poisoned by the absorption of decomposed secretions from the heated inflamed genital tract; they died because their exhausted vital forces were no longer able to bear up under the long continued depression of an unavailing labor. They died because of shock to heart and brain, caused by withholding the relief that should have been extended to a very dog!

"These opponents, with like seeming triumph, refer to the fact that one in every four or five of the children so delivered also perish. Of course they too perished, not because they were delivered with the forceps, but because the accursed doctrine of Denman withheld the life saving forceps until the children were already dead, or in a dying condition. Had the forceps been used as it should have been, hours before, the children and mothers might all have lived. The death of all these mothers and children is justly chargable, not to the forceps, but to its being withheld until too late. The cry of "meddlesome midwifery is bad" has been repeated from the days of Denman and Blundell to this, by every ignoramus who has sat for hours by the side of a helpless woman permitting her to suffer in ununavailing labor until her offspring was dead, and herself but little better. They died because the attending physician, through a criminal ignorance, was unable to give the necessary assistance, or, through blind adherence to an age of ignorance, he feared to do so, and remained trusting in the resources of nature long after these had shown themselvas inadequate to safe delivery, then the long deferred assistance came too late. Thanks to the march of science, as we predicted twelve years ago, the days of this ignorance have been fully numbered." (?)

" Perhaps the most triumphant vindication and argument for the frequent and early use of the forceps, and their utter harmlessness in skillful hands, is given in the practice of Dr. Hamilton, of Falkirk, who, though utterly ignorant of the mechanism of natural labor, upon a knowledge of which all truly scientific operations must be based, and with quite an imperfect instrument compared to Hodge's forceps (Zegler's straight forceps), and with this applied as it never should be, with blades antero-posteriorly, delivered one hundred and ninety children with only two deaths, or a little over one per cent. None of the mothers were lost. We hold it as a rule of practice to use the forceps in any case, in every case, when in the second stage of labor the head fails to advance during the pains, or advancing, does so so slowly that the labor threatens to be prolonged, to the injury of mother and child, without waiting for this injury to occur. More than this, we carry the war into Africa by freely declaring and teaching that the physician who permits his patient to linger unnecessarily in the second stage of labor when he could prevent it by the timely use of the forceps, is reprehensible in that he thereby endangers the well-being of those entrusted to his care. I use the forceps in every three or four cases, and have never regretted having used it even in a single case, but have often regretted that I had not used it earlier." "I have applied the forceps some twenty times at the superior strait. In two of these cases the head was not engaged or fixed in the inlet or brim, and had to be held down by an assistant. One of the children in these twenty cases was known to be dead before the forceps were applied, and one was lost by delay in delivering the shoulders after the head was delivered. In some eight of these cases the os was not dilated more than two inches. None of the children perished because of the forceps delivery. All the mothers did well. I have never delivered a woman with the forceps who died from, by, or in connection with her accouchment, and consequently have most certainly killed none with the instrument."

The above quotations are given from one of the ablest and most experienced obstetric teachers, as a fair specimen of modern teaching on that subject in our best medical schools. And yet, strange to say, we find men eager to take upon themselves the duties of accoucheur who utterly ignore all this, and blindly refuse their suffering patients the best, the safest and most easily applied means of relief that the inventive genius of man has bequeathed to suffering woman.

I hold that it is the duty of every one whose education embraces a thorough knowledge of these subjects to come out boldly and speak and act for the good of humanity. I hold that the obstetric forceps, in the hands of a well educated physician, is a perfectly safe and harmless instrument, and that its more frequent use would lead to a corresponding reduction of the sufferings and dangers of childbirth, and rescue thousands of the fairest and most interesting of our race from disease and suffering incident to protracted and difficult labors. Who can doubt that nine-tenths of the cases of uterine and vesical diseases in child-bearing women have been brought on by unnecessarily protracted labors? We all know the dangers incident to prolonged and intense muscular effort—how it loads the blood with effete material, exhausts the vital powers, and deranges every physiological function of the whole system.

And yet, how many delicate women are permitted to labor for hours, and sometimes for days, enduring the strain upon their muscular and nervous systems of the most intense effort, and at the same time enduring the agonies of pain the like of which is never experienced in any other condition of human existence. Is it any wonder, then, that disease and, in many cases, death should follow in the wake of such suffering when protracted beyond the limits of nature's power of endurance? And this point may be, and often is, reached while the *do-nothing* practitioner is endeavoring to console his patient with promises of speedy delivery. And I do not hesitate to say that in a large majority of such cases, the labor might and should have been terminated with the forceps in time to have prevented all danger and useless snffering.

Such is the mission of an instrument that is at once a pain-saving, a life-saving and a health-preserving means in the hands of the conscientious and skillful accoucheur. I commend it to the careful study and more frequent use of my professional brethren, whose duties so often bring them in contact with cases requiring its employment.

Induce your neighbor to subscribe for DANIEL'S TEXAS MEDICAL JOURNAL. $2.00 per annum, in advance.

